# Supplementary material for: Estimating SARS-CoV-2 seroprevalence and epidemiological parameters with uncertainty from serological surveys
Source: eLife. 2021 Mar 5;10:e64206. doi: 10.7554/eLife.64206 (PMC7979159; doi:10.7554/eLife.64206)
Supplement: Supplementary file 1. — Sensitivity and specificity values were taken from manufacturer’s claims as of February 2021 as filed with the U.S. Food and Drug Administration, 2021. [file elife-64206-supp1.pdf]

Manufacturer	Test	Sensitivity	Specificity
*EuroImmun	SARS-COV-2 ELISA (IgG)	90.0% (27/30)	>99.9% (80/80)
†Roche	Elecsys Anti-SARS-CoV-2 S	96.6% (225/233)	>99.9% (5990/5991)
†Abbott	Architect SARS-CoV-2 IgG	>99.9% (88/88)	99.6% (1066/1070)

* included only in main text modeling, calculations, and figures.

† included in Supplementary modeling, calculations, and figures.

Table 1: **Serological tests and performance characteristics considered in this study.** Sensitivity and specificity values were taken from manufacturer's claims as of February, 2021 as filed with the U.S. Food and Drug Administration [30].
