# Supplementary material for: Estimating SARS-CoV-2 seroprevalence and epidemiological parameters with uncertainty from serological surveys
Source: eLife. 2021 Mar 5;10:e64206. doi: 10.7554/eLife.64206 (PMC7979159; doi:10.7554/eLife.64206)
Supplement: Supplementary file 2. — This table is divided into four sections. The top two sections correspond to the parameters of the single-population modeling. The bottom two sections correspond to the parameters used in the age-structured modeling. Sections corresponding to θ and θi are separated to indicate that their values were used in synthetic data experiments to assess performance of the Bayesian inference methods on varying seroprevalence levels, to separate them from SEIR model parameters. Contact matrices Ci⁢j used in this article were, in particular, those corresponding to the U.S., India, and Switzerland. Equations for models can be found in the Appendix. Test kit sensitivity and specificity values are provided in Supplementary file 1. Subpopulation seropositivity values θi were synthetically generated to accommodate moderate variation between subpopulations as well as the ability for mean seropositivity to be easily adjusted (used in Figure 3, or were generalized to 5-year age bins from the age-stratified serosurvey of Stringhini et al., 2020) based in Geneva, Switzerland, as shown (used in Figure 5). [file elife-64206-supp2.pdf]

Single-population seroprevalence estimation			
Parameter	Meaning	Value	Notes
θ	Seropositive fraction in synthetic data experiments	various, between 0.01 and 0.50	used in Figure 2

Single-population SEIR model			
Parameter	Meaning	Value	Notes
α	Transition rate $E \to I$	0.2	
β	Infectiousness	1.75	
γ	Recovery rate	0.5	
ρ	Social distancing effects	0.5, 0.75	
(n_+, n_-)	(positive, negative) test counts	$(13, 87)$, $(130, 870)$	used in Figure 4

Age-structured seroprevalence estimation			
Parameter	Meaning	Value	Notes
θ_i	Seropositivity of age-i subpopulation in synthetic data experiments	$\tilde{\theta}$ + [-0.014, -0.012, -0.004, 0.002, 0.008, 0.015, 0.018, 0.020, 0.006, 0.005, 0.003, -0.003, -0.009, -0.010, -0.012, -0.012], with $\tilde{\theta}$ between 0.05 and 0.50, representing the unweighted average seroprevalence across subpopulations.	used in Figure 3
θ_i	Seropositivity of age-i subpopulation adapted from empirical study	[0.018, 0.018, 0.107, 0.107, 0.109, 0.109, 0.109, 0.109, 0.109, 0.109, 0.0821, 0.082, 0.082, 0.047, 0.047, 0.047]	[32]; used in Figure 5

Age-structured SEIR model			
Parameter	Meaning	Value	Notes
u_i	Relative susceptibility of age group i	[0.40, 0.40, 0.38, 0.38, 0.79, 0.79, 0.86, 0.86, 0.80, 0.80, 0.82, 0.82, 0.88, 0.88 , 0.74 , 0.74]	[27]
y_i	Probability that an infection is clinical for an age-i individual	[0.29, 0.29, 0.21, 0.21, 0.27, 0.27, 0.33, 0.33, 0.40, 0.40, 0.49, 0.49, 0.63, 0.63, 0.69, 0.69]	[27]
C_{ij}	Number of age-j individuals contacted by an age-i individual per day	U.S.A., India, and Switzerland	[18] (see caption)
μ_E	Mean incubation period	3 days	[27, 43]
μ_p	Mean duration of preclinical infectiousness	2.1 days	[27, 43]
μ_c	Mean duration of clinical infectiousness	2.9 days	[27, 44]
μ_s	Mean duration of subclinical infectiousness	5 days	[27]
f	Relative infectiousness of subclinical cases	50%	[27]

Table 2: **Parameter values used in dynamical models and numerical experiments.** This table is divided into four sections. The top two sections correspond to the parameters of the single-population modeling. The bottom two sections correspond to the parameters used in the age-structured modeling. Sections corresponding to θ and θ_i are separated to indicate that their values were used in synthetic data experiments to assess performance of the Bayesian inference methods on varying seroprevalence levels, to separate them from SEIR model parameters. Contact matrices C_{ij} used in this manuscript were, in particular, those corresponding to the United States of America, India, and Switzerland. Equations for models can be found in the Appendix. Test kit sensitivity and specificity values are provided in Supplementary File 1. Subpopulation seropositivity values θ_i were synthetically generated to accommodate moderate variation between subpopulations as well as the ability for mean seropositivity to be easily adjusted (used in Figure 3, or were generalized to 5-year age bins from the age-stratified serosurvey of Stringhini et al. [32] based in Geneva, Switzerland, as shown (used in Figure 5).
